# How heuristics in judgement influence the securities investment decision process

**DOI:** 10.1057/s41264-022-00184-7

**Published:** 2022-10-10

**Authors:** Marcel Piotrowski, Christian Bünnings

**Affiliations:** FOM – University of Applied Sciences, Essen, Germany

**Keywords:** Affect heuristic, Anchor heuristic, Availability heuristic, Cognitive psychology, Decision-making, Securities investments, Financial advice

## Abstract

Heuristics and biases are a widely researched topic in the field of behavioral finance. Yet, most studies only investigate the effects of heuristics and biases under laboratory conditions and consequently do not consider that financial decisions are likely to be made quite differently under laboratory conditions than in real life. Therefore, the purpose of our study is to investigate the effects of heuristics on the securities investment decision-making process in real-life financial advice conversations. To this end, we analyze the buying behavior of 200 individually observed retail customers of a German bank. Specifically, fifteen securities advisors were instructed to actively induce heuristics to measure how these cognitive biases distort customers' decision making with respect to purchase probability, one-time investment amount, and monthly savings plans. Our results indicate that heuristics in judgement affect the likelihood that customers purchase a product at all, but they do not seem to affect the amount of investment or the level of savings plans.

## Introduction

How do people judge what is right or wrong? How do people judge the value of their properties? How do people assess whether certain prices are too high or too low? Or how do people judge whether they should follow investment recommendations of their financial advisor or not?

Heuristics in judgment and decision-making aim at reducing complexity in various decision situations and are subject of an extensive body of scientific literature on cognitive biases. The origin of heuristics and biases can be attributed to Tversky and Kahneman (1974), who made a seminal contribution to cognitive psychology with their research about the availability, anchor, and representativeness heuristic. Since their pioneering findings, heuristics have been explored in a wide variety of contexts, including (behavioral) finances.

Chen et al. (2017) study the “January effect,” an anomaly in the stock market, and find that bonus payments for Taiwanese employees, which are usually paid before Lunar New Year, affect the demand for stocks and thus stock prices. Jordan and Kaas (2002) analyze heuristics with respect to mutual fund advertising strategies and observe the anchor heuristic to have a significant impact on perceived return expectations and the representativeness heuristic to influence the perception of risks. Other studies continue to consider emotional aspects and provide evidence that investment decisions are rarely made rationally but rather under the influence of cognitive biases (Kahneman & Riepe 1998; Young & O’Neill 1992). Jordan and Kaas (2002) and Shah et al. (2018) indicate that even well-experienced investors are regularly influenced by heuristic principles.

Although heuristics and biases in the context of financial decision-making are well established in the literature, most studies are conducted under laboratory conditions (Jordan & Kaas, 2002; Ackert et al., 2010). These studies show that people regularly rely on heuristic principles when making financial decisions, but do not consider that financial decisions, particularly investment decisions, are likely to be made differently in a laboratory than in real life. Michaelidou and Dibb (2008) indicate that consumer involvement affects how consumers make purchase decisions. While involvement is probably lower in a laboratory setting, it is rational to assume that involvement increases when customers’ real money is affected. This holds particularly true for financial investment decisions, where people typically invest significant amounts of their own money.

The objective of this paper is to follow up the findings by Lavin et al. (2019), who show that consumers regularly encounter difficulties when investing in securities and, as a result, make their investment decisions under the influence of behavioral biases. They observe participants to be guided by contextual information, such as marketable annual expense ratios, or to evaluate an investment fund’s quality in relation to the expense ratio of previous investments. While Lavin et al. (2019) analyzed how investors are guided by biases under laboratory conditions, the present paper analyzes the effects of heuristics in real-life financial consulting sessions based on the reasons described above.

This paper contributes to the existing literature on heuristics in the context of behavioral finance by analyzing how heuristics in judgment influence the securities investment decision process in financial consulting. To the best of our knowledge, there is no study on how heuristic principles influence the likelihood of purchasing investment products or the amount of investment in real-life financial advice conversations. In this context, the present paper examines the effects of the affect heuristic, the anchor heuristic, and the availability heuristic, considering how customers are guided by these heuristics in real financial consulting meetings. We restrict our analyses to these heuristics since they seem to play an important role in the context of securities advice in *retail* banking as outlined in more detail in the next chapter.

## Background and related literature

In general, heuristics are known as cognitive illusions that can distort people’s ability to make rational decisions and judgments under uncertainty (Kahneman & Riepe 1998). Heuristic principles are thereby applied to reduce the complexity of predictions, evaluations, and probabilities. While they are helpful principles in reducing complexity of various decisions and assessments, they can lead at the same time to systematic errors in judgement (Tversky & Kahneman, 1974). Heuristics in judgement and decision-making usually occur unconsciously in uncertain decision situations, while they can also be used to influence decision processes, such as a customer’s purchase decision in financial investments. In the context of this paper, heuristics are considered as a kind of tool for advisors to consciously guide their clients through investment decisions.

Since Tversky and Kahneman (1974) groundbreaking findings about heuristics and biases, various heuristics and biases have been explored in different financial contexts, such as the disposition effect describing the phenomenon that investors tend to sell stocks that have increased in value too fast, while holding stocks that have lost in value too long (Shefrin & Statman, 1985), the home bias relating to the fact that many investors are hesitant to invest in foreign equities (French & Poterba, 1991), or the overconfidence bias in investors’ trading skills (Statman et al., 2006). As outlined in the introduction, in this study we focus on the affect heuristic, the anchor heuristic, and the availability heuristic in the context of financial advisory meetings for private investors without extensive financial knowledge. We restrict our analyses to these heuristics since we expect them to play an important role in the context of financial advice to private clients as we outline in more detail in the following paragraphs. In addition, the selected heuristics can be integrated into securities advice without requiring complex preparations, i.e., advisors can easily integrate them into their everyday advice.

Mussweiler and Strack (2000) show that the anchoring and adjustment heuristic influences decision makers by anchor and reference points. In general, the literature indicates that people tend to rely on the first information they receive and make their financial decisions dependent on it (Shah et al., 2018). While the anchor heuristic was originally tested by Tversky and Kahneman (1974) in terms of estimates for mathematical equations, Lavin et al. (2019) investigate its impact on the willingness of retail customers to invest in specific investment products. Lacking complete information on quality and prices, investors make their investment decisions depending on predetermined contextual information, i.e., fictitious offer prices and a fictitious average market price, which serves as anchor value. In fact, subjects were more willing to invest in those products whose fictitious offer price was smaller than the fictitious average market price (anchor).

Besides the anchor heuristic, we expect the affect heuristic to influence investment decisions of retail customers. The rationale behind this idea is that money has a high emotional relevance and emotions are likely to influence the investment behavior of lay investors. Luo and Subrahmanyam (2019) find the affect heuristic to influence investment decisions and even well-being. That is, people who care about the environment might buy shares from companies that offers appropriate products, such as cars from Tesla Motors. Moreover, they observe a positive relationship between owning socially responsible stocks and people’s physic well-being. In the context of financial consulting, many customers follow the investment recommendations of their advisor only by relying on their emotions and gut feeling (Young & O’Neill, 1992). This holds also true for professional investors, who are biased in a similar way as lay investors (Jordan & Kaas, 2002). Financial advisors who understand emotional aspects and attitudes of their customers are able to build a trusting relationship and may elicit affect. In this context, Monti et al. (2014) show that advisors can use two main channels to evoke affect: spoken words and unspoken messages. This suggests that advisors can focus on affective language and use words and phrases that evoke positive affect in their clients instead of complex technical terms.

Choosing the availability heuristic is based on Slovic’s et al. (2007) suggestion that the affect heuristic is directly linked to the mere-exposure effect (Zajonc, 1968). The mere-exposure effect states that repeated exposure or presentation of a particular stimulus increases the likelihood that people will like that stimulus. When people are biased by the availability heuristic, they make decisions and judgements based on the ease with which information and thoughts can be brought to mind (Tversky & Kahneman, 1974). The latter, in turn, is closely related to the frequency of being exposed to a certain information or stimulus. Leynes and Addante (2016) provide evidence that those stimuli which have already been perceived can be decoded and processed more easily and quickly. This also applies to stimuli in the form of information about financial products. Another example of a stimulus in the context of investment decisions might be the investment behavior of nearby people (Shah et al. 2018). As a result of applying the availability heuristic, investors may face poor returns because of ignoring important investment parameters.

Against the background of the existing literature, we form three hypotheses, each divided into three sub-hypotheses.

### H1a

The anchor heuristic has a positive impact on purchase probability.

### H1b

The affect heuristic has a positive impact on purchase probability.

### H1c

The availability heuristic has a positive impact on purchase probability.

### H2a

The anchor heuristic has a positive impact on one-time investments.

### H2b

The affect heuristic has a positive impact on one-time investments.

### H2c

The availability heuristic has a positive impact on one-time investments.

### H3a

The anchor heuristic has a positive impact on saving rates.

### H3b

The affect heuristic has a positive impact on saving rates.

### H3c

The availability heuristic has a positive impact on saving rates.

Hence, we hypothesize all three heuristics to positively influence the buying behavior among private customers. Unfortunately, the literature provides no clear indication whether some heuristics may have a stronger influence on the purchase probability than others.

## Data and methods

To investigate the relationship between the heuristics described above and the investment behavior of individuals, a study was conducted in a German bank (retail customer advisory service) in the Rhine-Ruhr region (Germany, North-Rhine Westphalia) from January 2021 to April 2021. Fifteen financial advisors with securities competence level 1^1^ (according to the German WpHG) who advise private customers were selected for this study. The advisors were randomly drawn into three different groups, one for each heuristic. Furthermore, a control group was created with additional five, randomly selected advisors who did not learn about this study or the analysis of their consulting sessions. Each advisor conducted ten securities consultations, which were subsequently evaluated in terms of whether a product was purchased, the amount of one-time investments, and, if available, the amount of a monthly savings plan, resulting in 200 individual observations.

Depending on the group, the advisors were instructed to conduct usual securities advisory discussions with their clients, considering the individual study instructions given at the beginning of the study. The advisors only learned about the heuristic to which group they were drawn to. Hence, it is convincing that they only employed the heuristic described accordingly. These instructions were compiled carefully considering the existing literature and are briefly outlined in the following.

*Affect heuristic*

People who are biased by the affect heuristic associate a distinct affect with a particular event. Affect influences cognitive information processing and thus the customers’ purchase decisions. To trigger affect, advisors should use certain phrases that directly aim to evoke emotions. That is, the advisors were told to place emotional or affective purchase arguments according to the needs of their customers. They focused three commonly known buying motives: liquidity, security, and return. For example, the consultants used phrases such as “Your securities investment will make you very happy when you realize high returns” or “With this investment, you will feel very secure because you are investing in many different real assets”.

*Anchor heuristic*

Since people regularly make their decisions in relation to a specific anchor value, the advisors in this group were asked to set exactly one anchor point in relation to the deposit volume or savings plans in their advisory meetings. They were free to choose the level of the anchor as long as they were clear about the appropriateness of the anchor: If the reference point were set too high, customers could be deterred and thus negatively influenced in their decision. For example, advisors told their customers: “On average, my customers save EUR 130.00 a month in equity funds. Is that the same for you?”.

*Availability heuristic*

The advisors in this group focused on customers who aimed to invest in securities for the first time. In line with the results described above, they provided their customers with product information in advance: About 14 days before the advisory meetings, the advisors sent their clients a fund portrait by mail or e-mail. In the counseling sessions, they then referred to this mail or email to induce the cognitive availability bias. In addition, advisors were informed that this procedure is compatible with the MiFID II guidelines (Markets in Financial Instruments Directive II) for financial instruments.

## Data analysis and results

To estimate the relationship between the heuristics and the investment behavior of individuals, we set up the following basic linear regression model

$$y_{i} = \alpha + \beta\,{\text{heuristics}}_{i} + \gamma X_{i}^{\prime } + \varepsilon_{i}$$

with subscript *i* denoting customers and $\varepsilon_{i}$ the regression error term. The vector *heuristics*_*i*_ contains a set of dummy variables indicating the heuristics under scrutiny and the control group, respectively. In our main specification, the dependent variable $y_{i}$ is the decision to invest in a security product or not. Hence, we essentially estimate a linear probability model. However, in two further specifications $y_{i}$ represents the amount of one-time investments and the monthly savings rate, respectively. An additional dummy variable indicating whether a customer invested or not enters these specifications to not capture the effect of a potentially higher/ lower share of investors among the heuristic groups.

The coefficient vector of interest in all specification is $\beta$, representing the effect of different heuristics on the respective outcome variable. $X_{i}$ contains a set of socioeconomic controls (*age*, *gender* as well as *wealth* and *income in thousand euros*), which might confound the estimation results. Against the background of a small sample size ($n = 200$) and to adjust standard errors accordingly, we rely on bootstrapping methods with 10,000 replications.

Table 1 shows descriptive statistics of the entire and unconditional estimation sample as well as conditional on the respective heuristic group. Already here it is noticeable that in all three heuristic groups the frequency of product purchases (between 0.66 and 0.74) is consistently higher than in the control group (0.34). These frequencies give a first indication that there seems to be some influence of heuristics on the decision to invest in security products of customers. Although this holds also true for both the amount invested and the saving rate, the lower values in the control group might partly be the results of the lower investment rate in this group.

**Table 1:** Descriptive statistics

	Unconditional		Affect Heuristic		Anchor Heuristic		Availability Heuristic		Control Group	
	Mean	Min	Mean	Min	Mean	Min	Mean	Min	Mean	Min
	SD	Max	SD	Max	SD	Max	SD	Max	SD	Max
*Dependent variables*										
Product Purchase	0.61	0	0.66	0	0.74	0	0.7	0	0.34	0
	0.49	1	0.48	1	0.44	1	0.46	1	0.48	1
One-time Investment	7551	0	11,683	0	9760	0	6248	0	2512	0
	17,899	130,000	23,804	130,000	20,912	130,000	13,884	70,781	7439	36,000
Savings rate (mth.)	47.17	0	37.1	0	70.9	0	57.9	0	22.8	0
	73.24	500	56.91	300	105.34	500	58.91	225	50.78	200
*Control variables*										
Age	53.85	18	58.52	19	53.62	20	47.6	18	55.66	23
	18.48	86	17.94	86	20.32	84	17.96	81	16.24	85
Female	0.46	0	0.46	0	0.5	0	0.4	0	0.5	0
	0.5	1	0.5	1	0.51	1	0.49	1	0.51	1
Male	0.54	0	0.54	0	0.5	0	0.6	0	0.5	0
	0.5	1	0.5	1	0.51	1	0.49	1	0.51	1
Wealth (TEUR)	57.36	0.026	66.56	4.14	65.81	2.29	42.93	0.038	54.13	0.026
	46.05	165.22	47.18	162.71	47.3	165.22	41.13	138,61	45.56	164,20
Income (TEUR)	2.4	0.22	2.59	0.25	2.46	0.63	2.19	0.22	2.35	0.26
	1.12	7.02	1.2	5.81	1.22	6.67	1.1	7.02	0.92	4.75

Own calculations based on 200 observations

Considering the socioeconomic control variables, the distribution across the four groups indicates that randomization works well, as all control variables have similar means and standard deviations. This implies almost equal conditions for the advisors of the individual groups.

Table 2 comprises the empirical results of the main model that explains customers’ inclination to buy a security product. All estimated coefficients of the three heuristics are highly statistically significant and show the expected positive sign, indicating that using heuristics in an advisory discussion for security investments increases the likelihood of investing in such products. Hence, the estimation results support the hypotheses H1a, H1b, and H1c. The absolute magnitudes of the estimated coefficients are sizeable and highly economically relevant. To be more precise, the affect heuristic increases the purchase probability by around 34 percentage points, the anchor heuristic by around 40 percentage points, and the availability heuristic by around 32 percentage points. Compared to the unconditional purchase probability in the sample of 61 percent, this translates into an increase of more than 50 percent. The estimated effect size is even larger, when the estimated coefficients are related to the purchase probability in the control group, which is 34 percent. Although we observe some difference in the absolute magnitude of the estimated coefficients, these differences are statistically not significant as the confidence intervals are overlapping. Finally, we observe no significant relationships between the socioeconomic controls, in particular wealth and income, and the dependent variable, indicating the randomization indeed worked well.

**Table 2:** Linear probability model—purchase probability

	Estimated Coefficient	S.E.
Constant	0.6769 ***	0.1427
Affect heuristic	0.3355 ***	0.0936
Anchor heuristic	0.3966 ***	0.0948
Availability heuristic	0.3182 ***	0.0962
Age	− 0.0033	0.0020
Female	− 0.0893	0.0698
Wealth	0.0002	0.0009
Income	− 0.0488	0.0344
*R*^2^	0.142	
Observations	200	

Own calculations. Standard errors are based on 10,000 bootstrapping resamples

Significance codes: ***p < 0.001; **p < 0.01; *p < 0.05

In the next step, we investigate whether the heuristics applied in the advisory discussions are also related to the one-time amount invested or amount saved monthly, respectively. To account for the higher purchase rates in the three heuristic groups, an additional dummy variable indicating whether a product was bought or not (*purchase*) enters the models. The results of this exercise are depicted in Table 3. The variable *purchase* is positive and statistically significant, which is not surprising, since the dependent variables are zero if *purchase* takes on the value zero. In contrast to our main model, we observe no statistically significant relationship between the heuristics and the one-time amount invested. That is, the present results do not support hypotheses H2a, H2b, and H2c. However, the coefficient of the affect heuristic seems to influence one-time investments slightly, at least from an economic perspective. The lack of statistical support also holds for hypotheses H3a, H3b, and H3c as we observe no statistical significant relationship between the estimated coefficients of the heuristics and the monthly saving rate.

**Table 3:** Linear regression model: one-time amount invested and monthly saving rate

	One-time amount invested		Monthly saving rate	
	Estimated Coefficient	S.E.	Estimated Coefficient	S.E.
Constant	− 2166.7	3169.5	− 22.78	15.44
Purchase	12,373.8 ***	1881.7	77.59 ***	7.90
Affect heuristic	3257.5	2703.0	− 12.41	9.56
Anchor heuristic	− 397.8	2556.2	14.81	13.38
Availability heuristic	321.4	2254.2	8.24	9.32
Age	− 149.2	76.6	− 0.29	0.30
Female	184.4	1861.4	11.95	8.85
Wealth	216,7	53.4	0.04	0.13
Income	− 1,295.4	946.8	11.73 **	4.67
R^*2*^	0.357		0.315	
Observations	200		200	

Own calculations. Standard errors are based on 10,000 bootstrapping resamples

Significance codes: ***p < 0.001; **p < 0.01; *p < 0.05

While the heuristics are positively related to the likelihood that a customer invest at all, they seem to play no role for the absolute amount. Except for the control variables wealth and income, we find no significant relationship between the socioeconomic controls and the two dependent variables. Interestingly, we observe wealth to be significantly related to the one-time amount invested and income to be significantly related to the monthly saving rate. This has an intuitive appeal, as wealth is probably the main determinant for a one-time investment while income is the relevant measure for a monthly commitment like a saving rate.

## Discussion and conclusions

This paper aims to follow up on the findings of Lavin et al. (2019), which show that customers regularly make investment decisions under the influence of behavioral biases. While previous studies mainly examined effects of heuristics under laboratory conditions, this study analyzes the role of heuristics in real-life consulting sessions. More concrete, we investigate the influence of the affect, anchor, and availability heuristic on the probability to invest, the one-time investment level, and the monthly savings rate level.

We observe all three heuristics to be positively and statistically significant related to customers’ probability to buy an investment security. Actively triggering one of the three heuristics increases the probability of product purchase by around 50 percent as compared to the unconditional purchase probability. In addition, there is some indication that the anchor heuristic is the most influential bias, which is consistent with the findings of Lavin et al. (2019)—the anchor heuristic does indeed appear to influence customers’ investment behavior. However, we cannot rule out equal influences of all three heuristics due to overlapping confidence intervals. Conditionally on purchasing an investment security, we find no statistically significant influences between the three heuristics and the one-time amount invested and the monthly saving rate, respectively. These results indicate that heuristics in judgement affect the likelihood that customers purchase a product at all, but they do not affect the amount of investment or the level of savings plans.

Although this paper provides valuable insights into the role of heuristics on financial decision making, the study has also limitations, which in turn might serve as a guide for future research. First, the estimation sample size is small. Future research might use a larger number of customers over a longer period of time. This would—among other things—allow to gain interesting insights into the interplay of the heuristics applied and socioeconomic variables like age or gender by interacting the heuristics with these variables. In addition, a larger sample size would put more credibility on the estimated coefficients.

Second, this study focused on three selected heuristics. A follow-up study might consider additional heuristics, such as the home bias (French & Poterba, 1991) or the representativeness heuristic (Tversky & Kahneman, 1974), and, hence, could further clarify the role of heuristics in investment decisions in general.

Third, the data were collected during the COVID-19 pandemic, which represent an exceptional uncertain time. This holds true especially regarding investment decisions and might partially affect the results. However, the fact that data on our control group were collected during the same period of time might counteract potential confounding effects, at least to some degree. Moreover, our results are likely to represent lower bounds on the effects of heuristics on financial decisions since it is convincing that investors behaved particularly conservatively during this uncertain period.

Fourth and most importantly, we cannot rule out that the present results might be partially explained by the study design itself. That is, the consultants in the heuristics group might be more motivated than the consultants in the control group because they knew they were taking part in this experiment and thus might be aware that the success of their consultations might be analyzed. This, however, is the drawback of a study conducted in a real-life setting. Future studies could attempt to eliminate this limitation by comparing the sales success of consultants before and after such a study or by considering the sales success of other products as comparison group. Nevertheless, we are confident that our empirical results are not solely the consequence of this behavior. The main reason is that the consultants do not have an incentive for sales because there exist no financial incentives, nor were the results reported to their managers. Although the present results are consistent with the findings of previous studies, the absolute magnitude of the estimated coefficients should be interpreted with some caution.

## Appendix 1: Study instructions: affect heuristic

### Introduction

Thank you for your assistance in collecting data for this scientific study: How Heuristics in Judgment Influence the Securities Investment Decision Process. Below is a brief introduction to the objective of this research, the process, and individual introductions for your securities consultations.

### Heuristics in judgement and decision-making

The aim of this scientific investigation is to analyze how heuristics in judgment influence the investment decision process. Heuristics can be understood as cognitive biases, or as a kind of filter that is placed over a decision situation and thus influences decisions. A simple example will show you how easily decisions can be biased by heuristics. Please imagine a simple coin toss. You flip a coin, and you see heads (H) or tails (T). Now you are asked to indicate which of the following two outcomes is more likely:

1. H H H H
2. T H T H H

You probably tend to estimate the second result as more probable. But this intuitive assessment is simply wrong: The second result must be less probable because there is one more roll. As a result, the probability of outcome (2) must be less than the probability of outcome (1). Heuristics influence human decisions when flipping a coin, in the supermarket, or when advising on securities.

### Research objectives

The aim of this scientific study is to determine how heuristics influence the investment decision process in securities consulting. For this purpose, you will be randomly assigned to one of three different heuristics groups. Here you will receive concrete suggestions and examples of how you can incorporate heuristic principles into your advisory discussions. By and large, you can conduct your advisory sessions as usual, but you will additionally consider the instructions described below. You will also be asked to conduct exactly ten securities advisories that will be evaluated. In view of the quality of the study, please do not talk to members of the other groups about their heuristics.

### Data collection

You do not have to evaluate or collect any data yourself; the evaluation is done by the author of this study. After your consultations, please report the customer identification number and whether a product sale has taken place. All other information is taken from the consultation protocol.

### Affect heuristic

All fifteen counselors were randomly assigned to one of the three heuristics. You were assigned to the affect heuristic. People who are influenced by the affect heuristic have pronounced affective likes or dislikes toward an event. In this context, affect determines cognitive information processing, i.e., the decision. Concrete arguments for or against a product are judged to be more convincing if they are congruent with the affective, i.e., emotional, evaluation. Simply put, the affect heuristic thus generates judgment errors and cognitive biases in decision making under emotion. With this heuristic, you don't need to do anything else before the customer meeting. Just prepare for your conversations as usual.

During the customer meeting, I ask you to build buying arguments for a customer significantly on emotions. To do this, it is important that you find out your customer's individual motive for buying and build an emotional argument on this. The main motives for buying securities are—as I'm sure you know—availability, security, return or cooperation with the advisor. Your goal is to find out this motive in concrete terms. Below you will find an overview with exemplary arguments which you can use to convince the customer. Of course, you can add your own ideas to these examples as long as you use emotional arguments. Please adapt my examples to your individual formulations and preferences—this is the only way to be authentic. In order to carry out the experiment successfully, please introduce at least one affective = emotional argument in every customer meeting. Please note that underlined words are key words which must remain in the statements in their meaning.

**Table:**

Purchase motive	Example emotional argument
Liquidity	"Investing in this fund will feel very flexible to you because you can draw down the investment whenever you want"
	"Your investment in securities will feel very flexible to you because you are very well protected with your other reserves (financial cushion, nest eggs)"
Security	"With this investment, you will feel very secure because you are investing in many different tangible assets"
	"Your securities investment will feel very safe to you because you are investing in a diversified portfolio"
Return	"You will be very happy with this investment because you will receive high increases in value through the reinvestment of earnings"
	"Your securities investment will make you very happy because you can realize very high opportunities through the company investments"
Cooperation	"You will be very happy with your investment because you can always reach me as a contact person"
	"You will be very happy with your investment because you have a strong partner for your securities transactions in the Sparkasse"

## Appendix 2: Study instructions: anchor heuristic

1–4: Cf. “Appendix 1”.

### Anchor heuristic

All fifteen consultants were randomly assigned to one of the three heuristics. You were assigned to the anchor heuristic. Under the influence of the anchor heuristic, people make a decision based on a predetermined anchor, usually a number. If you go to a bakery on Saturday to get your rolls and they charge you €0.30 [anchor] per roll, you will be shocked when another baker charges you €1.30 on Sunday. Another example is buying a car—usually the seller deliberately sets the price well above the market price. The buyer then offers a price well below the market price and eventually they meet in the middle. Studies have shown that anchors work even when they are not causally related to the decision.

You don't need to consider anything before the customer meeting with this heuristic. In the customer meeting, please place exactly one anchor. The following overview gives you some examples that you can use. Of course, you can expand these examples with your own ideas—the only important thing is that you place exactly one anchor. Additionally, it is important that the anchor is set as high as possible—but also not too high, otherwise you will "scare" your customer. The following overview will certainly give you a good feel for which anchors are appropriate. When in doubt, just listen to your gut feeling.

"Mr. X, the other day I saw that my customers' deposits contain an average of €74,020.00!"

"Mrs. Y, on average, my customers save €129.50 per month in equity funds. Does that match up for you as well?"

"Mr. Z, I'll just show you how you should structure your investment using the example of €100,000.00."

## Appendix 3: Study instructions: availability heuristic

1–4: Cf. “Appendix 1”.

### Availability heuristic

All fifteen consultants were randomly assigned to one of the three heuristics. You were assigned to the availability heuristic. People influenced by the availability heuristic give higher weight to information if it is readily available (i.e., easily retrieved from memory). Under the availability heuristic, people rate the likelihood of a traffic accident occurring higher if they learned about a traffic accident shortly beforehand through the media. In the same way, in the supermarket, in front of the shelf with ten different detergents, you would be more likely to reach for Lenor if you had recently seen Lenor advertisements on TV.

It is important with this heuristic that the customer is influenced with a short impulse before the meeting. Therefore, please send your customers monthly fund information of an investment fund that is not yet included in the customer's portfolio without comment at least 14 days before the meeting. For example, if the customer has Deka-DividendenStrategie CF and Deka-GlobalChampions CF in his portfolio, send him the monthly fund information of Deka-Industrie 4.0 CF in advance. You are completely free in your choice of funds, provided you select a product that is not yet included in the customer's securities account. This procedure is MiFID II-compliant and therefore in order from a regulatory point of view.

## Footnotes

1 In German banks, not every advisor is allowed to advise on securities investments. In the bank considered in this study, advisors can achieve four securities competence levels: With competence level 1, they are allowed to advise selected mutual funds; with competence level 2, they are allowed to advise a more comprehensive selection of mutual funds; with competence level 3, they are allowed to advise stocks and bonds (as direct investments); with competence level 4, they are allowed to advise all types of securities.
